# Adapting Community Detection Algorithms for Disease Module Identification in Heterogeneous Biological Networks

**DOI:** 10.3389/fgene.2019.00164

**Published:** 2019-03-13

**Authors:** Beethika Tripathi, Srinivasan Parthasarathy, Himanshu Sinha, Karthik Raman, Balaraman Ravindran

**Affiliations:** ^1^Department of Computer Science and Engineering, Indian Institute of Technology Madras, Chennai, India; ^2^Initiative for Biological Systems Engineering, Indian Institute of Technology Madras, Chennai, India; ^3^Robert Bosch Centre for Data Science and AI, Indian Institute of Technology Madras, Chennai, India; ^4^Department of Computer Science and Engineering, Ohio State University, Columbus, OH, United States; ^5^Department of Biomedical Informatics, Ohio State University, Columbus, OH, United States; ^6^Department of Biotechnology, Bhupat and Jyoti Mehta School of Biosciences, Indian Institute of Technology Madras, Chennai, India

**Keywords:** overlapping community detection, non-overlapping community detection, disease module identification, biological networks, heterogeneous networks

## Abstract

Biological networks catalog the complex web of interactions happening between different molecules, typically proteins, within a cell. These networks are known to be highly modular, with groups of proteins associated with specific biological functions. Human diseases often arise from the dysfunction of one or more such proteins of the biological functional group. The ability, to identify and automatically extract these modules has implications for understanding the etiology of different diseases as well as the functional roles of different protein modules in disease. The recent DREAM challenge posed the problem of identifying disease modules from six heterogeneous networks of proteins/genes. There exist many community detection algorithms, but all of them are not adaptable to the biological context, as these networks are densely connected and the size of biologically relevant modules is quite small. The contribution of this study is 3-fold: first, we present a comprehensive assessment of many classic community detection algorithms for biological networks to identify non-overlapping communities, and propose heuristics to identify small and structurally well-defined communities—*core* modules. We evaluated our performance over 180 GWAS datasets. In comparison to traditional approaches, with our proposed approach we could identify 50% more number of disease-relevant modules. Thus, we show that it is important to identify more compact modules for better performance. Next, we sought to understand the peculiar characteristics of disease-enriched modules and what causes standard community detection algorithms to detect so few of them. We performed a comprehensive analysis of the interaction patterns of known disease genes to understand the structure of disease modules and show that merely considering the known disease genes set as a module does not give good quality clusters, as measured by typical metrics such as modularity and conductance. We go on to present a methodology leveraging these known disease genes, to also include the neighboring nodes of these genes into a module, to form good quality clusters and subsequently extract a “gold-standard set” of disease modules. Lastly, we demonstrate, with justification, that “overlapping” community detection algorithms should be the preferred choice for disease module identification since several genes participate in multiple biological functions.

## 1. Introduction

Biological networks, such as protein–protein interaction networks, gene regulatory networks, gene co-expression networks, metabolic networks, signaling networks provide a mathematical representation of biological systems. In this work, we are interested in the study of networks that encode interactions among proteins. These interactions can be *physical*, where proteins bind to one another, or *functional*, where proteins are associated with one another for performing a particular task. Analyzing biological networks is essential for guiding biological experiments—these experiments could otherwise be very difficult to perform, or even intractable, if every gene or protein were to be characterized individually.

Biological networks have been observed to be highly modular (Hartwell et al., 1999), where a tightly connected group of genes (nodes) are involved in similar biological functions. These groups are referred to as communities, modules, or clusters. Modules detected from biological networks are usually responsible for a common phenotype and are useful in providing insights pertaining to biological functionality. Module identification methods (also known as community detection methods) play a crucial role in obtaining these functional modules.

Disease phenotypes are usually caused by the malfunctioning of certain genes, these group of genes is referred to as *disease module*. As genes responsible for a phenotype often possess common functionality, there exists a strong association between disease modules and functional modules (Goh et al., 2007; Zanzoni et al., 2009; Barabási et al., 2011). We know that the modular structure of the biological network is often useful in identifying functional modules; so, we proceed with the assumption that the same would be useful to identify disease modules. It is essential to identify these disease modules, as it could be helpful for various applications, such as the comprehensive molecular understanding of the disease, identification of co-occurring diseases, or the identification of extensive set of genes for targeted therapies.

**Present Work**. Various algorithms exist in the literature for community detection (module identification). Many are evaluated on *in silico* generated benchmark networks (Friedman et al., 2001; Girvan and Newman, 2002; Newman, 2006). However, performance of these multitude of community detection approaches across variety of these biological networks to discover biologically relevant modules (disease modules or functional modules) remains poorly understood. Such a diverse set of biological networks are fundamentally different owing to the generative processes underpinning their structure, it is important to evaluate performance of different approaches across them. In this work, we study the adaptability of these community detection approaches for disease module identification, notably in the context of the recent an open-community challenge called as the DREAM challenge (Dialogue for Reverse Engineering Assessments and Methods) on Disease Module Identification (DMI)^1^. The challenge posed the problem of predicting “non-overlapping” and small modules of size ranging from 3 to 100 nodes, across six different networks. The set of predicted modules from a community detection method were evaluated against 180 GWAS datasets to find out any significant association of modules with complex trait or disease, to identify disease modules amongst them.

We comprehensively assessed various existing module identification algorithms across diverse biological networks and propose novel algorithms with the notion of *core communities*, to identify small and structurally well-defined communities. We obtained a substantial improvement over the traditional approaches. To our concern, a common problem existed for all the non-overlapping clustering approaches—the number of enriched modules were quite less in comparison to the number of modules predicted. Also, the number of diseases enriching the modules were very less in comparison to the number of different GWAS datasets (180 GWAS datasets) available for testing. These observations beg multiple questions: (1) Does the disease module possess a community structure at all? (2) Could we build “ground-truth disease modules” whose structural properties could be analyzed? (3) Do all of the diseases have structurally well-defined modules associated with them? (4) Most importantly, is “non-overlapping” community detection suitable for disease module identification as in this challenge? (5) Lastly, is there any association between the diseases, in terms of common nodes in the community structure?

We address all of these questions in the present study. In summary, our main contributions are as follows:

- We have introduced a framework for *core module identification*, to identify small and structurally well-defined communities. We show that this is important to identify compact modules from biological networks, and to achieve a better performance in identifying disease-relevant modules.
- We report a comprehensive assessment of many classic community detection algorithms across 6 different types of biological networks, evaluated over 180 GWAS datasets. With our proposed approach, we achieved 50% performance improvement in identifying disease-relevant modules over classical approaches.
- We have also analyzed the patterns of connectivity in a disease module to better understand the properties of disease modules. We propose a method to identify *gold standard disease modules* based on the genes already shown to be associated with a particular disease.
- We show that *overlapping community detection* is a better approach for the identification of disease-relevant modules. Overlapping community detection is a preferred solution as a gene could be responsible for multiple diseases, and hence should be part of various disease modules.
- We have utilized *overlaps* of the disease modules, which are genes that are involved in multiple diseases (or disease module), to identify diseases that occur together, i.e., *co-morbid diseases*.

## 2. Materials and Methods

### 2.1. Data

In this section, we summarize the six different biological networks that were made available as part of the DREAM challenge. We have identified disease modules in each of these networks. We also introduce the Genome-Wide Association Study (GWAS) dataset that is central for evaluating the modules predicted by the community detection algorithms.

#### 2.1.1. DREAM Challenge Biological Networks

The organizers of the DMI DREAM challenge provided a unique collection of biological networks for humans. This collection included multiple physical interaction networks (protein interaction networks, signaling network) and functional gene networks (co-expression, homology, and cancer). The statistics on the number of nodes and edges in these networks are presented in Table 1. In this section, we will briefly describe these networks.

**Table 1:** Network statistics of different biological networks.

**Network type**	**#Nodes**	**#Edges**	**Edge Weight**	**Density**	**Clustering coefficient**
PPI-1	17,397	2,232,405	Confidence	0.01475	0.13759
PPI-2	12,420	397,309	Confidence	0.00515	0.12421
Signaling	5,254	21,826	Confidence	0.00133	0.00227
Co-expression	12,588	1,000,000	Correlation	0.01262	0.05209
Cancer	14,679	1,000,000	Correlation	0.00928	0.14288
Homology	10,405	4,223,606	Confidence	0.07803	0.04153

**Protein-Protein Interaction Network-1:** The human protein-protein interaction network-1 (PPI-1) data were obtained from STRING version 10.0 (Szklarczyk et al., 2014) after removing the interactions derived from text mining. In this network, the nodes represent proteins, and the edges represent interactions, with the weights representing confidence scores.

**Protein-Protein Interaction Network-2:** Similar to PPI-1, this is also a protein interaction network, obtained from InWeb (Li et al., 2017), where the interactions are aggregated from primary databases and literature. Again, the proteins are the nodes in the network, and their reported physical interactions are the edges. The edge weights represent the confidence in each interaction.

**Signaling Network:** Türei et al. (2016) have provided the signaling network, which represents signaling pathways. In this case, the nodes are the genes, and the directed edges between them represent the gene interactions responsible for a cellular function. The weights represent the confidence scores from the experiments that have reported the interaction. Genes in this network can be mapped to corresponding proteins in the other networks.

**Co-expression Network:** Co-expression network was obtained from Gene Expression Omnibus (Barrett et al., 2010) and captures the correlation between the expression patterns of genes. These expression patterns of genes are observed across various samples of the experiments performed under different experimental conditions. The network is created with genes as nodes and co-expression as the edge between them.

**Cancer Network:** The cancer network is derived from Project Achilles (Cowley et al., 2014), which performed experiments to determine tumor-wise essential genes that are critical for the survival of that tumor. Those genes that are essential and are absolutely necessary for a tumor to function are connected through an edge in the cancer network. These correlations between the gene expression patterns with respect to a tumor are studied across various tumor samples. The correlation scores obtained through these experiments are represented as edge weights.

**Homology Network:** The homology network is constructed by connecting genes which are evolutionarily related. Evolutionarily related genes were identified using the Clustering by Inferred Models of Evolution (CLIME) (Li et al., 2014) algorithm. The algorithm partitions the genes into sets of evolutionarily conserved module. The algorithm also provides the confidence scores based on the evolutionary evidence, which are represented as weights of the edges connecting the evolutionarily connected genes in the homology network.

#### 2.1.2. Pre-processing

Biological networks being noisy, pre-processing these networks plays an important role. We sparsified the networks by removing the edges with low weights. We removed edges having weights lesser than two standard deviations from the mean. This not only reduces computation time for the various approaches but also improves the performance of methods by reducing noise.

#### 2.1.3. Genome-Wide Association Study (GWAS)

A Genome-Wide Association Study is an observational study conducted across different individuals. The objective of the study is to investigate the association between genetic variants across the whole genome and traits. The genetic variants refer to the variations that occur in a nucleotide at any specific position in a genome. We have a comprehensive set of 180 GWAS datasets associated with various complex traits and diseases, which belong to broader categories of 15 diseases, as shown in Table S1. Modules predicted by the community detection algorithms are tested against each of these GWAS datasets.

### 2.2. Methods for Module Identification

This section details the various approaches that have been used in our experiments. Methods discussed under “Module identification using non-overlapping community detection” form the basis for our proposed framework, as we detail in section 2.3. Methods discussed under “Overlapping community detection” are primarily used to analyse the properties of disease modules, as discussed in section 3. The purpose of this section is to give an overview of the methods available for module identification in networks, which are leveraged by us to improve module identification in biological networks to identify disease modules.

#### 2.2.1. Module Identification Using Non-overlapping Community Detection

Non-overlapping community detection methods are frequently adopted in the biomedical research (Choobdar et al., 2018). However, such methods restricts every node in a network to belong to a single community, and due to extensive cross talk among various genes and pathways, this restriction in biological networks is untenable. To understand the performance of different module identification methods with such restrictions, we tried some of the most commonly accepted approaches in the field of biology such as modularity maximization (Newman, 2004; Blondel et al., 2008), Markov chain CLustering (MCL) (Dongen, 2000), and Community detection using External and Internal scores in Large networks (CEIL) (Sankar et al., 2015) across various biological networks. We now discuss various state-of-the-art approaches based on (1) quality measures to define community structure, and (2) random-walk based methods to identify community structure.

##### 2.2.1.1. Community quality measures

A network can be defined as G={V,E}, where V is a set of *n* nodes and E⊆V×V, is a set of *e* edges. The network are represented using an adjacency matrix *A*, which is square matrix of dimension |V|×|V|. The element *A*_*ij*_ in the matrix is zero when there is no edge between node *i* and node *j*, and non-zero representing the weight of the edges connecting the nodes; for unweighted networks the value is one. The degree of a node *i* in the network denoted as *d*_*i*_, is the number of edges from a node to the other nodes, i.e., $d_{i}=\underset{j\in V}{\sum }A_{ij}$. Next, we define some important network parameters that enable measurement of community quality.

**Modularity:** Modularity is defined for a group of nodes, as the difference between the number of edges between those nodes in the original network and a null model, which is essentially a random rewiring of the original network, retaining degree distribution. The higher the difference, the better is the connectivity between the nodes. For a good community the modularity score should be high. The highest value is one. Modularity for a community *c* is defined as follows:

(1) $$\begin{array}{l} Modularity\left(c\right)=\frac{1}{2e}\underset{\begin{array}{c} i,j\;i\neq j \end{array}}{\sum }\left(A_{i,j}-\frac{d_{i}d_{j}}{2e}\right)\delta_{c_{\left(i\right)}c_{\left(j\right)}} \end{array}$$

where $\frac{d_{i}d_{j}}{2e}$ represents the expected number of edges between nodes *i* and *j*, *c*_(*i*)_ represents the community to which node *i* belongs and

(2) $$\delta_{c_{\left(i\right)}c_{\left(j\right)}}=\{\begin{array}{ll} 1 & \text{if }{\text{c}}_{\left(\text{i}\right)}{\text{=c}}_{\left(\text{j}\right)}\$\\ 0 & \text{otherwise} \end{array}$$

Modularity based method for community detection prefers group of nodes with higher concentration of edges than expected as communities.

**Conductance:** Conductance is a measure to define the quality of the community, based on how well-separated the nodes in the community are to the rest of the network. It measures the cut of the community concerning the internal connectivity of the nodes in the network. A good community is isolated from rest of the networks thus have low conductance. The conductance of the community c is defined as:

(3) $$Conductance(c)=\frac{\Sigma_{i\in c,j\in \bar{c}}A_{i,j}}{min(InternalEdge(c),InternalEdge(\bar{c}))}$$

where $\bar{c_{i}}$ comprises of the rest of the network other than the nodes in *c*_*i*_ and,

(4) $$\begin{array}{l} InternalEdge\left(c\right)=\underset{i\in c}{\sum }\underset{j\in V}{\sum }A_{i,j} \end{array}$$

**CEIL:** Community detection using External and Internal scores in Large networks (CEIL) (Sankar et al., 2015) is another way of measuring the quality of the community. CEIL strikes the middle ground between modularity and conductance which takes into account: (1) the internal density of the community, and (2) the separability of the community from the rest of the network, measured by internal and external score respectively.

The density of the community is the ratio of internal community edges and possible edges inside the community. The separability of the community from the rest of the network is measured as the ratio of internal community edges and edges that are incident on that community. CEIL Score for a community *c* with *n*_*c*_ nodes is the product of internal and external score which are defined below.

(5) $$InternalScore(c)\;=\{\begin{array}{ll} \frac{InternalEdge(c)}{\left(\begin{array}{c} n_{c}\\ 2 \end{array}\right)} & \text{if }n_{c}>\text{1}\\ 0 & \text{if }n_{c}\text{=0} \end{array}$$

(6) $$ExternalScore(c)\;=\frac{InternalEdge(c)}{InternalEdge(c)+\Sigma_{i\in c,j\in \bar{c}}A_{i,j}}$$

(7) $$\begin{array}{lll} CEIL\left(c\right) & = & InternalScore\left(c\right)\times ExternalScore\left(c\right) \end{array}$$

##### 2.2.1.2. Markov Chain Clustering

Markov Chain Clustering (MCL) (Dongen, 2000) is a random walk-based approach. With the help of random walks, the flow of the network is analyzed and communities are located where the flow tends to gather. For MCL, two processes are alternated on the network, (1) *expansion*, which involves taking powers of the transition matrix to determine the flow of the network, and (2) *inflation*, which involves re-scaling and normalizing the columns and then taking the power of the column.

The application on real-world network of these methods could be found in the work of Fortunato (2010).

#### 2.2.2. Module Identification Using Overlapping Community Detection

Overlapping community detection allows a node to be part of multiple communities thus resulting in overlapping communities. As genes are commonly involved in multiple functionalities, we have explored overlapping clustering to identify disease modules. The overlapping clustering approaches that we have explored involve two phases to identify communities: (1) “seed node” selection and (2) seed expansion. Since seed node selection is the most critical step to initialize the communities, we have explored multiple strategies to identify nodes that are likely to be “disease nodes.” The phases of community detection are discussed below.

##### 2.2.2.1. Seed node selection

We now describe our approach to identify seed nodes, which forms the basis for our algorithm to predict overlapping communities.

**Disease seed nodes:** Considering the genome-wide significance threshold of 10^−4^ as defined by Choobdar et al. (2018), the genes having a *p*-value below this threshold were considered as disease seed genes. We also considered 10^−6^ as a threshold to keep a stricter constraint. We defined *disease seed nodes* as the set of genes that pass the threshold across the 180 GWAS datasets.

**Unsupervised seed nodes:** In the absence of information about known disease nodes, we find a correlation between disease genes and network centrality measures like degree centrality and clustering coefficient of nodes. We observed that disease genes have a higher degree in comparison to the non-disease genes. Consequently, we used HITS (Schütze et al., 2008) and spread hubs (Whang et al., 2016), which are based on the degree of a node, as a seed selection mechanism, to select some important nodes from the network. We grow the communities using PPR scores as described in Andersen et al. (2006). As there is no information involved about the disease seed nodes, we call this process as *unsupervised seed node*.

##### 2.2.2.2. Seed expansion

**Figure 1:**
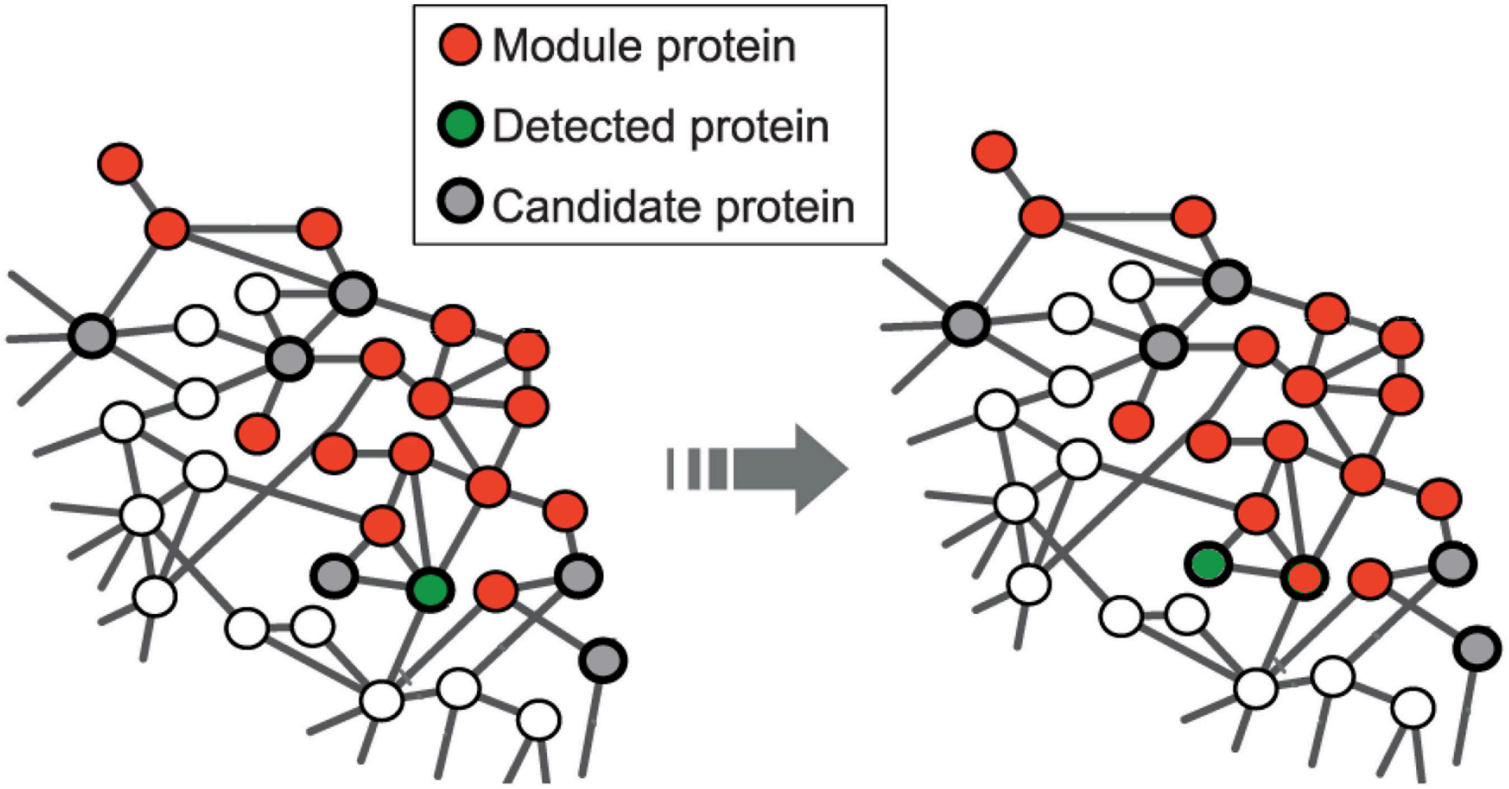
Red nodes represent the module formed by disease seed genes; the set of potential candidate nodes in the local neighborhood of the module is shown in gray; the green node represents the detected gene based on the personalized page rank score and will be included in the module at the next step. This is adapted from the image licensed under the CC BY 4.0 license and attributed to Ghiassian et al. (2015). A DIseAse MOdule Detection (DIAMOnD) Algorithm Derived from a Systematic Analysis of Connectivity Patterns of Disease Proteins in the Human Interactome. PLoS Comput Biol 11(4): e1004120. https://doi.org/10.1371/journal.pcbi.1004120.

The seed expansion is done based on the Personalized PageRank (PPR) scores as described in Andersen et al. (2006). PPR scores are used to rank the nodes in the neighborhood of a seed node. The nodes, in the order of their ranking based on PPR scores, are added to the module one by one till the size of the set reaches a particular value (100 for us) as shown in Figure 1. The modularity score of the group is computed after the addition of every node. The group of nodes that has the maximum modularity among the different groups, obtained after each addition, forms a module. This seed node expansion process is done for all the seed nodes.

From next section onwards we will discuss about our proposed work.

### 2.3. Proposed Framework—Core Module Identification

Biological networks exhibit a power-law (Barabási and Albert, 1999) degree distribution, where a few nodes have very high degrees whereas most of the nodes have small degrees. Performing community detection on these networks results in a few giant communities corresponding to the high degree nodes, along with multiple small communities. These giant communities cover most of the network and are least informative to derive any biological insights. Thus, there exists a need to improve the setup to perform community detection. Works in the past, such as those done by Berger and co-workers Singh et al. (2006), take into account the domain knowledge for generating finer clusters. However, identifying finer clusters without any domain knowledge is an interesting problem to be studied. We have proposed few approaches in this section to obtain finer clusters.

We introduce the term *core of the module* to represent finer modules. A core is structurally the strongest part of the module. We have designed four different frameworks to extract the core module, which are explained below:

#### 2.3.1. Ensemble Approach to Clustering

There exist multiple topological definitions of communities and multiple metrics like modularity, conductance, etc. to identify them. However, which topological definition suits a “biologically meaningful” community, is not well-studied. It would be interesting to incorporate multiple topological aspects to generate biologically meaningful modules.

Asur et al. (2007) and subsequent followup (See surveys Ghosh and Acharya, 2013; Ji et al., 2014), suggest ensemble frameworks to combine different clustering algorithms on biological data. Many approaches are suitable for base clustering approaches, that have a fixed number of predicted clusters. Working with a fixed number of clusters might not be the best way of identifying communities from a network, as we do not know *a priori* the desired number of clusters. We develop a simple yet novel approach to compute a consensus from the approaches that do not require the number of clusters to be fixed.

**Figure 2:**
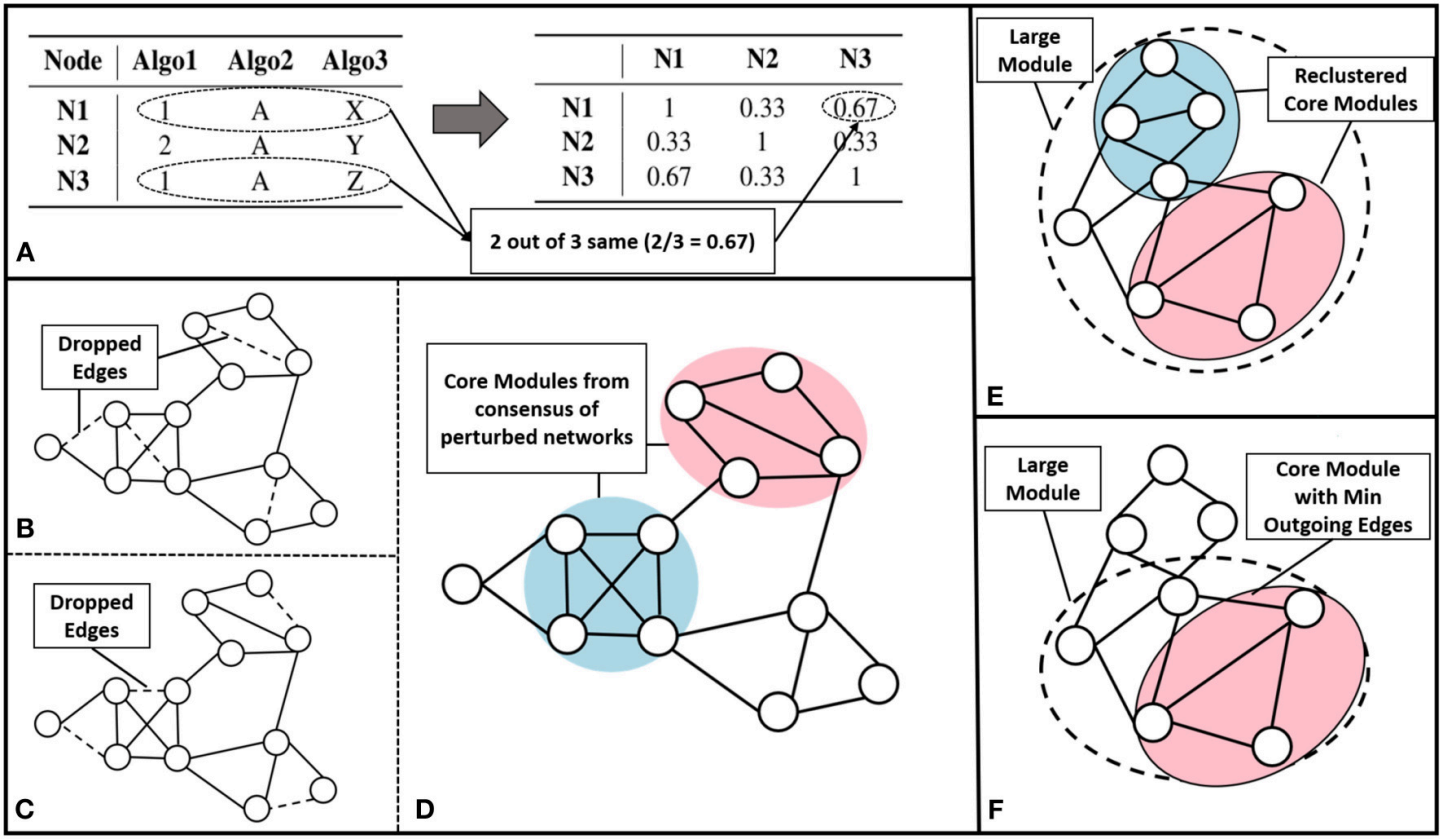
Core module identification methods **(A)** The process of *ensemble clustering*, which uses the power of multiple community detection approaches. A vector of community assignment is created for each node (left). The consensus is taken by computing the Jaccard similarity between the community assignment vectors, for every pair of nodes (right). **(B–D)** *Perturbation*, the process of perturbing the network and finding consensus module across the set of perturbed networks: **(B,C)** are examples of perturbed network after randomly dropping 1% of the edges (dashed lines), **(D)** community detected across the set of 100 perturbed networks. **(E)** *Multiple Core Identification* breaks the large module identified by a community detection algorithm into smaller modules as shown in the example where the dotted circle represents a large module and the colored circles represent the multiple cores obtained, by breaking down the larger modules. **(F)** From a large module *min outgoing edges* selects the group of nodes with minimum outgoing edges and maximum internal connection as in the example where dotted circle represents the large module and colored circle represents the core.

We have built an ensemble framework that takes consensus across various approaches, like modularity maximization (with different resistance parameter settings to obtain modules of different sizes), MCL and CEIL. These approaches captures varied aspects of the network structure without fixing the number of clusters to be predicted. We consider *r* base clustering approaches for a network with a set of nodes $V={\left\{v_{i}\right\}}_{i=1}^{n}$. We build a vector for each node, **v**, {[*v*]_*q*_| *q* = 1, 2, ..*r*}, where each element corresponds to the community assignment of that node in the *qth* clustering algorithm (see Figure 2A left).

The pairwise Jaccard similarity (Jaccard, 1901) between nodes, represented as $J_{sim}\{v_{i},v_{j}\}=\frac{\left\|\{v_{i}\cap v_{j}\}\right\|}{\left\|\{v_{i}\cup v_{j}\}\right\|}$, is computed to obtain the similarity between the community assignments across all the nodes (as demonstrated in Figure 2A right). For example, if the similarity between a given pair of nodes is unity, it means that the nodes co-occurred in the communities predicted by all the algorithms. We then built a similarity matrix out of these pairwise Jaccard similarity values, and subsequently constructed a network from this similarity matrix by linking the nodes having a similarity greater than 0.5. Finally, we use modularity maximization to perform module identification on the resultant network.

#### 2.3.2. Perturbations to Identify Robust Communities

Biological networks have a lot of inherent noise (Bader and Hogue, 2002), caused by the incompleteness of data or experimental biases. Therefore, it is important to identify communities that are robust to noise in the network. To identify robust communities, we follow a setup of perturbing the network multiple times and then performing a community detection on the perturbed networks.

We perturbed the network by randomly dropping 1% of edges. We repeated this for 100 iterations as indicated in Figures 2B,C. To detect communities on all the perturbed networks, we follow a setup similar to the ensemble approach described earlier, performing modularity maximization on the similarity network. This enabled us to identify modules *persistent across* perturbed networks. The process is explained in the Figure 2D.

#### 2.3.3. Core With Minimum Outgoing Edges

A community should have a higher number of edges connecting the nodes within a community (“internal edges”) (Newman, 2004) and a fewer number of edges connecting nodes outside the community (“outgoing edges”) (Kannan et al., 2004). For large communities, we identify a core that consists of the nodes that satisfy the property of a good community. These are the nodes that have a higher number of internal edges and a fewer number of outgoing edges. To this end, we have computed a *core score* for each node *n*, which considers the ratio of outgoing edges to internal edges from that node as follows:

(8) $$\begin{array}{l} CoreScore\left(n\right)=\frac{OutgoingEdges\left(n\right)}{InternalEdges\left(n\right)} \end{array}$$

**Figure 3:**
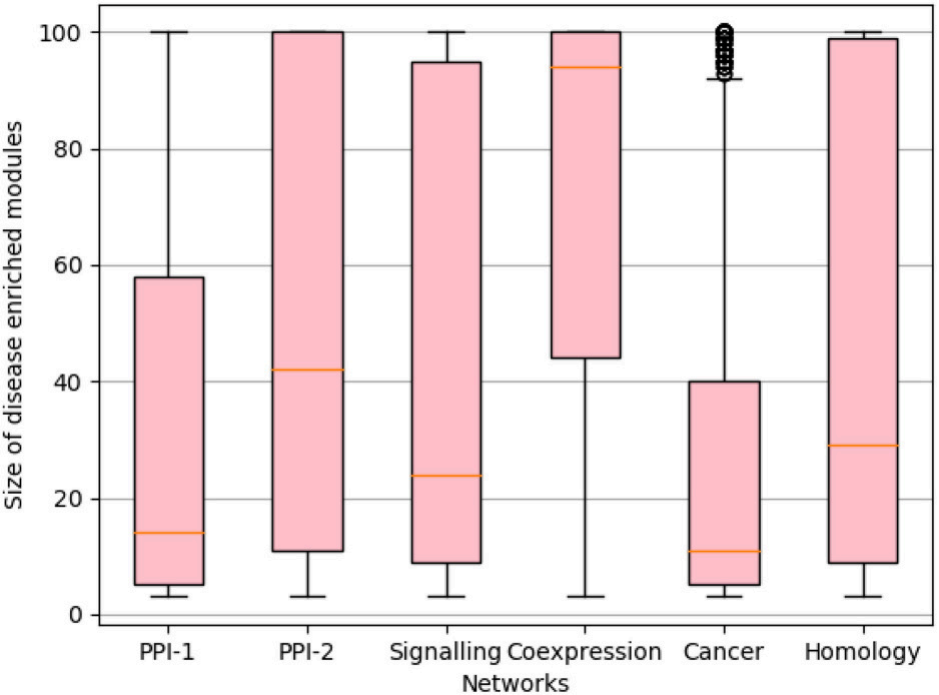
Size distribution of disease-enriched modules identified by various methods across networks. The X-axis represent different network and Y-axis represents the size distribution of disease enriched modules. The orange line in the box-plot represents the mean of the distribution and bubbles represents the outlier data points.

We rank the nodes in a module on the basis of their core score, i.e., nodes with lower scores get better ranks. In the case of larger communities of size more than 100, we consider the top 60 nodes as the core and ignore the remaining nodes (Figure 2F). We consider only top 60 nodes as we figured out through empirical studies by running multiple experiments that the average size of a disease module is 60. Figure 3 shows the size distribution of the disease modules obtained using multiple approaches. This approach helped in pruning the least important nodes from modules.

#### 2.3.4. Multiple Core Identification

We defined an iterative way of performing community refinement. In the first step, we used modularity maximization to identify modules in the network. Some of the resulting modules can be quite large due to the high connectivity of few of the nodes. There is a higher chance of occurrence of multiple well-connected cores in a single large module, as depicted in Figure 2E. However, it is difficult to avoid merging of these modules at the time of module formation process during the modularity maximization step. Generally, modules grow quickly around a high-degree node due to the frequent merging of communities around it, whereas modules grow slowly around the section of a network having low-degree nodes. If we stop the iterative module formation early, to capture smaller communities, it often compromises on the module lying in the sparser regions of the network. Therefore, we allow the module formation step to progress until there is no change in the modularity score of the entire network. Thereafter, we perform an iterative partitioning of larger modules into multiple smaller modules. This re-clustering resulted in many smaller modules fitting the size requirements of the challenge.

### 2.4. Overlapping to Non-overlapping Community Assignment

For understanding the sensitivity of the overlapping and non-overlapping clustering approaches, we convert the overlapping communities to non-overlapping communities and compare their performance. Initially, we form a *base community*, which is comprised of only those nodes that got a single community assignment. To obtain non-overlapping communities the nodes that are part of multiple communities, i.e., the overlaps of the communities, needs to be assigned to one of the base community. We have suggested the following three ways of assigning the overlap to the base community:

**Random Allocation:** Random allocation involves assigning the nodes in the overlap randomly to one of the base communities. The drawback of this method is that the community structure is not well-defined after the assignment.

**Conductance Assignment:** In order to maintain the structure of a community, the node assignment should be based on some quality measure for the community. We have used conductance. We assign nodes in the overlap to the base community with which it has the minimum conductance score.

However, while assigning a node to a base community, the conductance score for each node is independently checked against each base community. This means that, toward the end of the node assignment, the community structure need not be preserved, as all the nodes were independently assigned and the inclusion of even a single node can drastically change the community structure.

**Iterative Conductance Assignment:** To resolve the problem addressed in the previous approach, we follow an iterative way of assigning the node to the base community. Each node in the overlap is assigned to the base community with the minimum conductance, one after the other, and the conductance score is re-computed for the base community. This a called as a phase of community assignment.

After completion of a community assignment phase, the nodes which were part of the overlap are extracted from the base community one by one and reassigned to the community with which it has the best conductance score. This is done to avoid any bias due to the order in which the nodes were assigned. Thus, the phases are repeated iteratively till convergence, when no node changes its community. Though we do not give a proof of convergence, we have empirically observed that this approach converges after a few (typically 3–5) iterations.

### 2.5. Evaluating Disease Modules

The DREAM challenge organizers provided a novel framework for assessing the methods based on the number of predicted modules that are significantly associated with complex traits and diseases (with the help of GWAS data). Instead of using traditional methods that take into consideration the functional annotation or pathway databases, they used GWAS datasets. This methodology of scoring is better, unlike functional annotations that originate from a similar type of functional genomics as the networks themselves. GWAS provides an entirely orthogonal view, for validation.

#### 2.5.1. Module Scoring Using PASCAL

PASCAL (Lamparter et al., 2016) stands for PAthway SCoring ALgorithm, which is a tool used to integrate SNP-trait associated *p*-values to incorporate gene-score and module score as illustrated in Figure S1. The gene score is computed by aggregating SNP-*p*-values for a GWAS dataset while correcting for confounders such as Linkage Disequilibrium (LD) correlation structure as explained in Figure S1A. For the module genes which are in LD and cannot be treated independently, this fast gene scoring method fuses the genes and recomputes the gene score as in Figure S1B. Modified Fisher method is used for computing enrichment in high scoring genes, where genes in the network become the “background gene set”. The **enrichment score** is defined as the number of modules with the significant score at 5% FDR (false discovery rate) cut-off for at least one of the GWAS dataset. The final score of the method is the number of disease enriched modules it discovers.

### 2.6. Implementation

All the approaches in core module identification, module identification using non-overlapping community detection and overlapping to non-overlapping community assignment were implemented in Python. For modularity maximization, we have used the implementation from the NetworkX package for Python (Hagberg et al., 2008). The MCL-edge software provided by Enright et al. (2002) was used for finding clusters using MCL. The implementation of CEIL algorithm was taken from the source code provided by Sankar et al. (2015). The evaluation script was provided by DREAM challenge organizers.

## 3. Results

Next, we study the community structure of the networks, to investigate if the disease modules are indeed clusterable, and proceed to answer the questions posed in section 1. We then show the importance of performing an overlapping community detection, and how it captures far more relevant modules. We further go on to illustrate how some knowledge of communities, in terms of “seed nodes” can positively impact the quality of clusters. Lastly, we show that overlaps of the disease modules helps in identifying comorbid diseases.

### 3.1. Core Module Identification Captures a Higher Number of Disease-Relevant Modules Than Traditional Community Detection Approaches

The well-known non-overlapping clustering approaches like MCL, modularity maximization and CEIL, tend to identify communities that are large and their size is dependent on the size of the network. However, disease modules are generally small. Wilber et al. (2009) have shown that small communities in these networks are biologically homogeneous. Biological homogeneity is evaluated from the functional similarity between pairs of genes, which is available from resources such as the Gene Ontology Database (Ashburner et al., 2000). They have shown that the functional similarity between pairs of genes in a small module is significantly higher than the functional similarity between all possible pairs. Core module identification methods identify smaller and structurally better communities. The size distribution of the traditional and core module based methods can be seen in the Figure S2.

Most of the methods that are considered have hyper-parameters; varying them could control the size and the number of modules detected. We have evaluated all the methods through an extensive grid search (parameter tuning) and report the best result for each method; the corresponding hyper-parameters are mentioned in the Table S2. For *MCL*, we vary the inflation (I) parameter in the range [2, 9] at intervals of 1 and the expansion is fixed at 2. The resistance (R) parameter for *modularity* is varied in the range [0.1, 1] at intervals of 0.1. *CEIL* does not have any hyper-parameter to be tuned. Table S3 presents the detailed results at each parameter setting. Core module identification methods are frameworks to extract compact modules and are built on top of the baseline methods. For core module identification, we have experimented with all the baseline methods and have reported the one's giving the best performance along with its hyper-parameter. The winners of DREAM challenge used Diffusion State Distance (DSD) (Cao et al., 2013, 2014) as a distance measure to perform kernel-based clustering. We have compared against their winning results. For the *perturbation* method, modularity maximization (with R as 0.1) is applied on all the perturbed networks; then consensus is taken over the modules predicted across perturbed networks. *Ensemble* uses all the baseline methods with all possible hyper-parameters. *Recluster* was done on the giant modules obtained from the best reported baseline method for that network. Therefore, the baseline method along with their hyper-parameter are reported in the table. The method for selecting nodes with *minimum outgoing edges* was applied after recluster method.

The results denote the number of enriched modules out of the predicted modules from the methods. The enrichment of a module is tested using PASCAL tool across 180 GWAS datasets. The results with best hyper-parameter setting are given in the Table 2; the number of disease-enriched modules identified by core module-based methods is much higher than those identified by the baseline approaches (Table 2A). In addition, we also show the “hit ratio” (Table 2B), illustrating the fraction of predicted modules that are enriched. Some methods, such as CEIL predict a large number of modules, but not many of them are enriched. On the other hand, our method, although it predicts marginally fewer modules, shows a much higher hit ratio. The reason for performance improvement on applying the proposed heuristics is due to the identification of core modules, which are smaller and structurally better, as discussed in section 2.3. From our proposed methods, min outgoing edges has the best performance with respect to number of disease-enriched modules identified, as it is a two-way refinement process (1) reclusters the giant modules obtained from baseline methods; therefore making the modules small with better internal connectivity (2) selects the nodes based on core score thus pruning away the less important nodes. The performance of our model is comparable to the winning team's performance, and in networks like PPI and Cancer, our method even outperforms the winning team's method, showing the strength of our model.

**Table 2:** Results of module identification approaches on simple networks, using off-the-shelf approaches mentioned as baselines, core module identification proposed by us and Diffusion State Distance (DSD) which is the winning method of DREAM challenge.

**Network**	**Baselines**			**Core module based methods**				**DSD**
	**MCL**	**Modularity maximization**	**CEIL**	**Perturbation**	**Ensemble**	**Recluster**	**Min outgoing**	
**(A)**								
PPI 1	16 (872)	8 (262)	12 (1398)	18 (260)	16 (250)	20 (460)	22 (462)	**24 (1020)**
PPI 2	18 (1125)	9 (209)	11 (1696)	17 (284)	17 (601)	19 (1311)	**21 (1311)**	19 (445)
Signaling	9 (268)	10 (111)	6 (320)	9(180)	8 (191)	11 (144)	14 (77)	**17 (194)**
Co-expression	9 (463)	10 (194)	5 (1336)	13 (126)	12 (202)	17 (145)	20 (205)	**24 (207)**
Cancer	4 (598)	4 (164)	5 (831)	6(598)	5(249)	3(518)	**9 (114)**	7 (329)
Homology	8 (180)	6 (177)	7 (320)	7 (168)	7 (87)	7 (212)	10 (149)	**11 (212)**
Score	64	47	46	70	65	77	96	**102**
**(B)**								
PPI-1	0.0183	0.0305	0.0086	**0.0692**	0.064	0.0435	0.0476	0.0235
PPI-2	0.016	0.0431	0.0065	**0.0599**	0.0283	0.0176	0.0195	0.0427
Signaling	0.0336	0.0901	0.0188	0.05	0.0419	0.0764	**0.1818**	0.0876
Co-expression	0.0194	0.0515	0.0037	0.1032	0.0594	**0.1172**	0.0976	0.1159
Cancer	0.0067	0.0244	0.006	0.01	0.0201	0.0058	**0.0789**	0.0212
Homology	0.0444	0.0339	0.0219	0.0417	**0.0805**	0.033	0.0671	0.0519

*The result contains, for different methods, **(A)** the number of enriched modules out of the total number of predicted modules in brackets. The score in the last row represents the sum of disease modules predicted across the six different networks, and **(B)** “hit ratio”, illustrating the fraction of predicted modules that are enriched*.

*The numbers in bold highlight the best approach for each of the 6 networks. The results show that DSD approach predicts more enriched modules, while core-module based approaches give higher hit-ratio (ratio of enriched to total predicted modules)*.

### 3.2. Clusterability of Disease Modules: An Analysis of Non-overlapping Community Detection

On analyzing the results of non-overlapping clustering approaches a common problem existed for all the methods, the number of enriched modules was quite less in comparison to the number of modules predicted. Also, the number of diseases enriching the modules were very less in comparison to the number of different (180) GWAS datasets available for testing.

**Figure 4:**
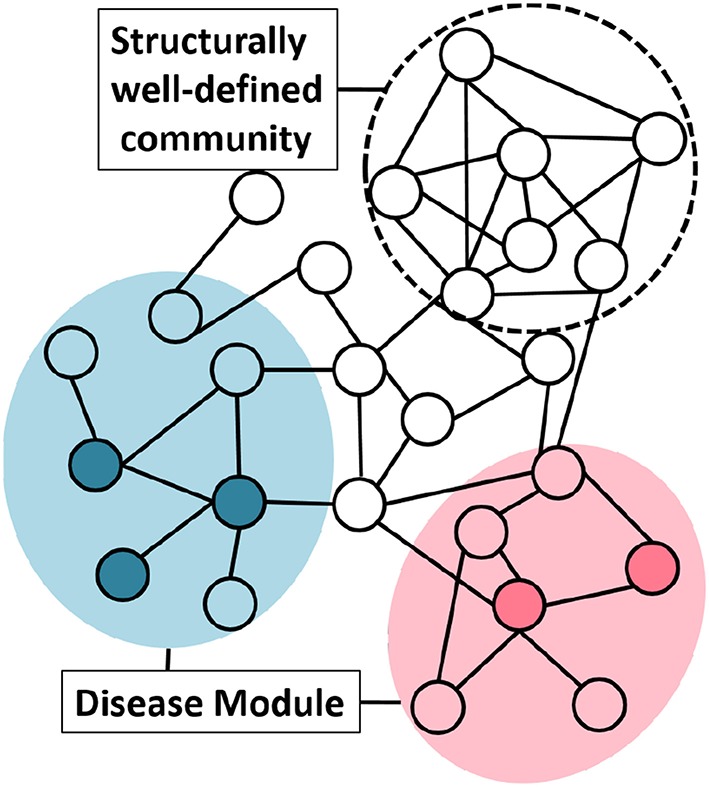
Group of genes associated with a disease do not necessarily possess graph-theoretic community structure. Nodes with the same color represent genes associated with the same phenotype. The shaded circle over the colored nodes represents the possible disease module while a “structurally well-defined community” need not be enriched with a specific disease.

To understand the network structure of disease module we studied the “clusterability” of disease modules. We define clusterability as the connectivity strength or the quality of the module. The ground truth disease modules are readily not available. To analyse the clusterability of a disease module, we try to understand the connectivity between the genes that are known to have an association with the same phenotype. These disease genes need not be highly interconnected to possess the graph-theoretic community structure. This phenomenon could be explained with the help of the Figure 4, where, the same colored nodes represents genes associated with a disease. As is evident from the figure, there are two possibilities: (1) genes associated to disease are in the neighborhood but are not so strongly connected to qualify the definition of community, or (2) structurally well-defined community need not be associated with a particular phenotype.

To understand clusterability, we examined the cluster quality of the largest connected component (LCC) of genes (nodes) in the network associated with the same GWAS dataset. Here, the cluster quality is defined on the structural properties of the cluster. We obtain cluster quality based on modularity and conductance scores. Modularity score is the difference between the number of edges that fall within the given clusters and the expected number of edges if edges were distributed at random (Newman, 2004). Whereas, conductance is indicative of dense connections within the group, and fewer links to the rest of the network. For good quality clusters, a higher modularity score (best is 1.0) and a lower conductance (best is 0.0) are preferred.

**Figure 5:**
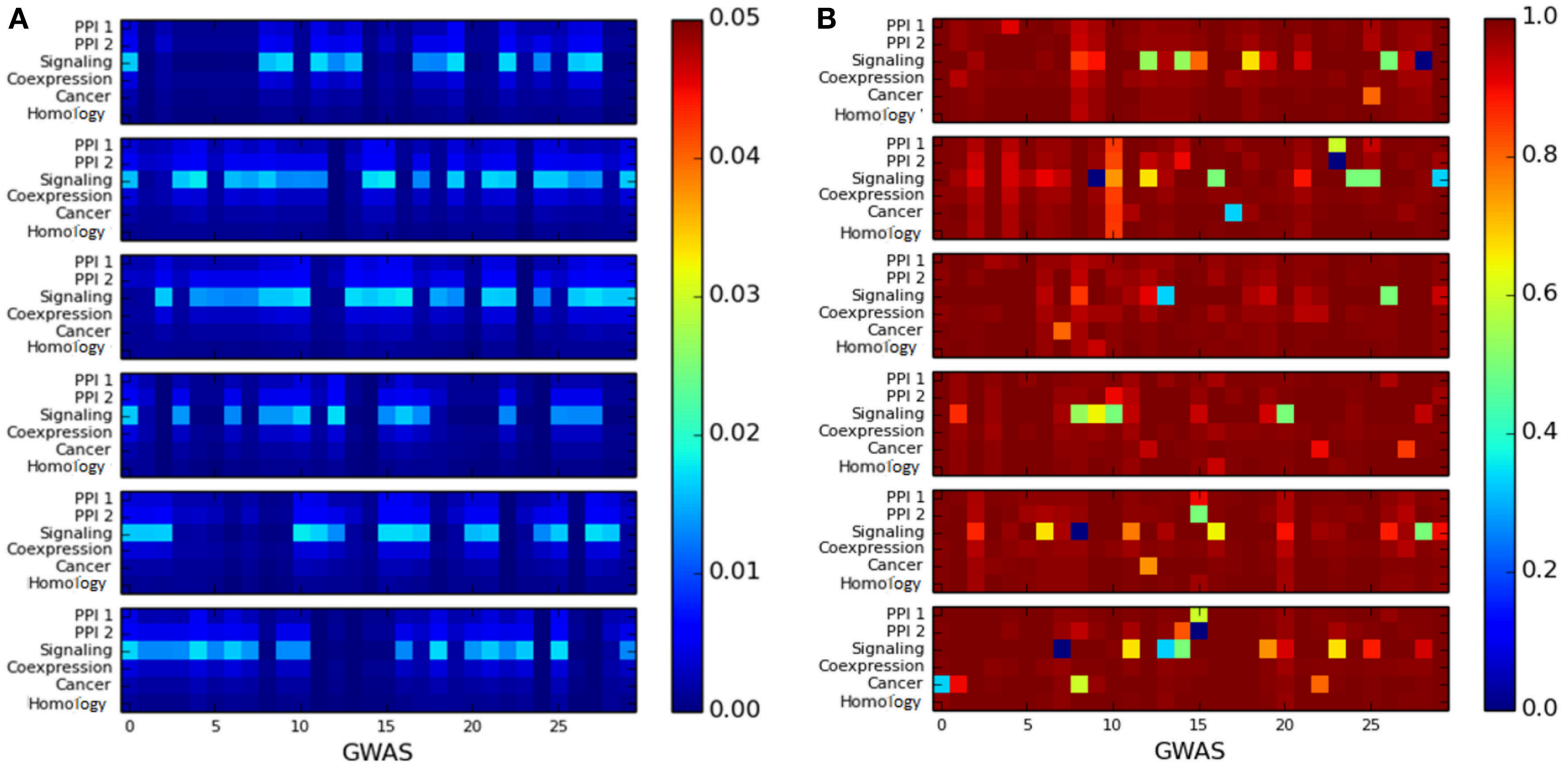
Heatmap for measuring the quality of the largest connected component of genes associated with a single disease using: **(A)** modularity score, where higher score is preferable and **(B)** conductance score, where lower score is preferable. The X-axis represents the 180 GWAS datasets and Y-axis represents the six networks. The color bar on the right represents the color coding for values marked in the heat map.

We observed that the cluster quality of the LCC of trait-associated genes is quite poor. The cluster quality of the LCC is depicted by the heatmaps representing as shown in Figure 5: the X-axis represents cluster quality across 180 GWAS datasets, which are stacked one above the other in groups of 30 datasets (stacking was done to aid visualization). The Y-axis represents the six networks. All the LCC have poor modularity scores, which are close to 0 as in Figure 5A. The conductance score is also poor for most of the LCCs, as shown in Figure 5B.

Community detection methods based on optimizing cluster quality measures fail to identify disease modules because of poor modularity and conductance scores of these modules. Therefore, it is hard to identify disease modules using community detection approaches based on optimizing these cluster quality measures. However, it would be interesting to study the structurally well-defined community that could be obtained from these LCC.

### 3.3. Approximating Gold Standard Disease Modules

The ground truth disease module are readily not available in order to substitute we identify structurally well-defined modules initiated from known disease-associated gene and define it as *gold-standard disease module*. We obtain the trait-associated genes from the 180 GWAS datasets and call them as *disease seed nodes*. We explicitly try to enforce the community structure into these groups by adding the neighborhood nodes using the seed expansion process.

#### 3.3.1. Gold Standard Modules Exhibit Clusterability

The modules obtained after this disease seed node expansion procedure were checked for enrichment using the PASCAL tool as described in section 2.5.1. The enriched communities so obtained are called as the *gold-standard disease modules*. These modules have proper community structure and are curated from the significant disease nodes. The statistics pertaining to the number of disease seed nodes obtained and the number of gold-standard modules identified are shown in Table 3. The percentage of the seed nodes covered in the enriched communities represents that these seed nodes have a well-structured disease neighborhood around them. “Disease spread” represents the number of GWAS datasets from out of 180 of them that could be identified in a particular network. The empirical results as in Table 3 suggest that many diseases have a good community structure in the PPI-1 network. These results also show that prior knowledge of disease seed nodes improves the performance of community identification by ten times as opposed to purely network driven community detection (Table 2). For example, in the case of PPI-1, we find 337 disease-enriched modules with this approach, compared to 22 from section 2.3.

**Table 3:** Gold standard disease modules identified from disease seed node expansion, keeping *p*-value threshold as 10^−4^ and 10^−6^ across 180 GWAS datasets.

**Network**	**Significance threshold 10^−4^**				**Significance threshold 10^−6^**			
	**# Seed nodes**	**Enriched**	**Disease spread**	**Seed nodes in enriched (**%**)**	**# Seed nodes**	**Enriched**	**Disease spread**	**Seed nodes in enriched (%)**
PPI 1	5436	337 (5433)	52	39.09	3103	266 (3101)	57	37.12
PPI 2	3876	130 (3844)	28	21.05	2267	103 (2250)	32	21.53
Signaling	1893	158 (1840)	36	31.80	1174	126 (1139)	44	37.39
Co-expression	4099	174 (4094)	34	53.86	2406	152 (2404)	38	54.61
Cancer	4507	6 (4429)	5	2.37	2555	2 (2522)	2	1.76
Homology	3227	28 (3154)	7	13.39	1861	14 (1826)	6	10.31

*The number of seed nodes obtained is mentioned in the 2nd and 6th column. The 3rd and 7th columns show the number of enriched modules against the predicted ones mentioned in the brackets. Disease spread represents the number of unique GWAS datasets identified across all the predicted modules. Percentage of seed nodes covered in the module is also tabulated*.

The disease seed node expansion procedure is helpful in identifying many disease enriched modules in comparison to the methods described in section 2.3. However, along with the increase in the number of enriched modules, there is also an increase in the number of non-enriched modules. We now study the difference in cluster quality of the enriched and the non-enriched modules.

We calculate the cluster quality of all the modules, predicted by the gold standard module identification process, using modularity and conductance scores. The predicted modules are divided into two sets—enriched and non-enriched modules—based on the enrichment predicted by the PASCAL tool (section 2.5.1). The distributions of cluster quality scores for the enriched and non-enriched modules across the six networks were then compared using notched box plots.

**Figure 6:**
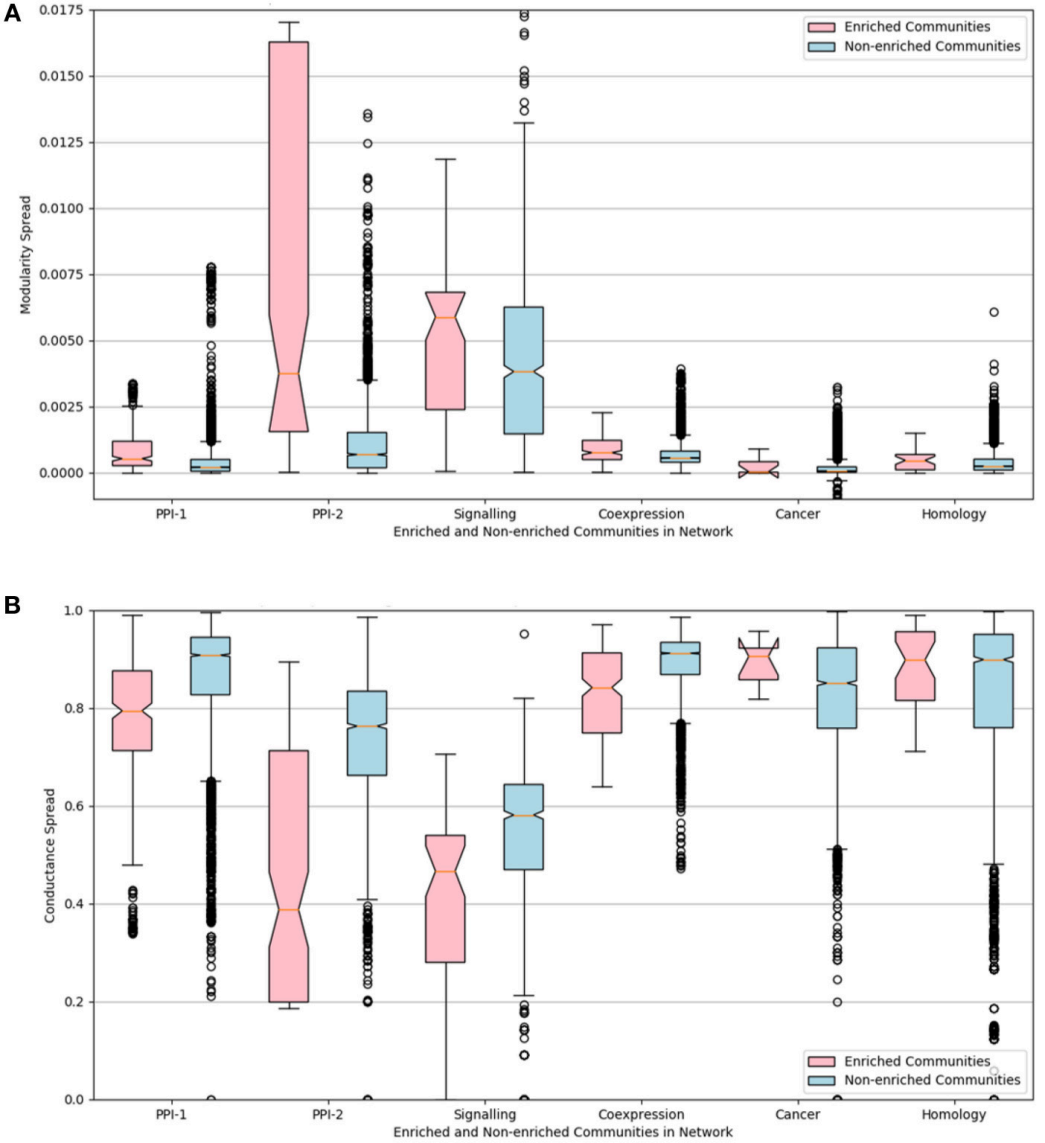
Notched box-plot representing **(A)** modularity and **(B)** conductance of enriched modules (pink) compared to non-enriched modules (blue) across the six networks. High modularity and low conductance is preferred for better quality clusters. Owing to the lack of overlap between the notches of the two distribution, the enriched modules have a significantly higher modularity and lower conductance score in comparison to non-enriched modules. **A notched box plot** is a graphical way of representing data. The box represents the interquartile range (IQR) of the data, where 50% of the data fall. The middle line denotes the median of the data. The top whisker is 1.5 times more than 75 percentile (Q3), and bottom whisker is 1.5 times lesser than 25 percentile. The notch represents the confidence interval around the median. The visual interpretation of these notches is that, if notches of box plot of two distributions do not overlap, then their medians differ with 95% confidence.

The distributions of the modularity and conductance scores of enriched and non-enriched modules can be visualized in the Figure 6. The notch represents the confidence interval around the median. The visual interpretation of these notches is that, if notches of box plot of two distributions do not overlap, then their medians differ with 95% confidence. The mean of the distributions of scores for enriched and non-enriched counterparts vary significantly as there is no overlap between the notches of the two distributions. This variation is quite significant for PPI-2 and signaling networks.

We can conclude from the study that disease-enriched modules in all the networks have better clusterability properties. So the predicted modules could be ranked on the basis of modularity or conductance scores, and the higher ranked modules are more likely to be disease modules.

#### 3.3.2. Amount of Disease Seed Nodes Required for Expansion

We took 10^−4^ and 10^−6^ as *p*-value thresholds, to identify disease seed nodes. The identified disease seed nodes across the set of 180 GWAS dataset is quite large in comparison to the total number of genes in the network as can be seen in Table 3. For example, in the case of PPI-1, 5436 disease seed nodes are identified, whereas there were 17397 genes in the network (from section 2.1), which means 30% of the network is a part of disease seed nodes.

The percentage of genes covered in the disease modules indicate that not all disease seed nodes are required for disease module identification. Here, we proceeded to analyse the amount of known disease seed nodes required for expansion, and how the performance is impacted knowing a fewer number of disease seed nodes. We randomly selected 10, 50, and 80% of the known seed nodes (with a *p*-value cutoff of 10^−6^) and performed disease seed node expansion from these and observed the enriched modules obtained. This step of randomly selecting *k%* nodes was repeated five times to avoid any bias due to a single run. The enriched modules reported in Table 4 shows the average of the modules predicted in five runs. It is observed that for some networks like PPI-1, PPI-2, and signaling, increasing the number of known seed nodes improves the number of disease modules recovered. In other networks, namely, homology, cancer and co-expression, the number of known seed nodes did not substantially change the number of disease modules identified.

**Table 4:** The number of modules predicted when starting with different fraction of initial known seed nodes (10^−6^).

**Disease Seed**	**PPI 1**	**PPI 2**	**Signaling**	**Co-expression**	**Cancer**	**Homology**
100% 10^−6^	266 (3101)	103 (2250)	126 (1139)	152 (2404)	2 (2522)	14 (1826)
80% 10^−6^	165.8 (2147.6)	90 (1357.2)	53.4 (597.6)	152.2 (1859.4)	5 (1484.2)	14 (1221.8)
50% 10^−6^	111 (1378.4)	60.6 (897.4)	35.2 (407.6)	105.8 (1167.4)	5.2 (979.6)	13 (793.8)
10% 10^−6^	40 (292.8)	20.6 (202.6)	16.8 (98.2)	29 (236)	8.4 (216.8)	12 (174.2)

*Here, the value represents the number of enriched modules out of the total number of modules predicted mentioned in brackets*.

### 3.4. Disease Modules Are Naturally Overlapping and Transcription Factors Mostly Lie in the Overlaps of Disease Modules

**Figure 7:**
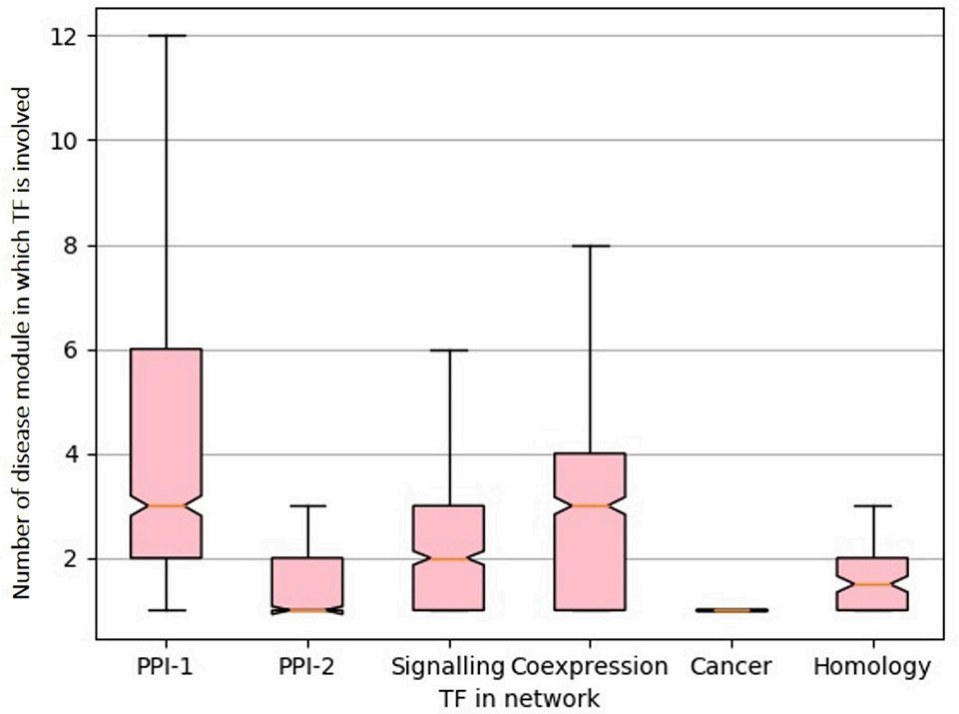
Box-plot representing transcription factors involved in multiple diseases. The X-axis represent different network and Y-axis represents distribution of number of disease module a TF is involved. The notches in the box-plot represents the mean of the distribution.

From the gold standard module identification procedure we obtain overlapping communities and the drastic increase of almost 10 times in the number disease modules identified in comparison to non-overlapping methods (Tables 2, 3) suggests that “overlapping methods” should be a preferred choice for disease module identification. We also try to find out the biological relevance for the nodes that are part of multiple communities. An *overlap* is defined as the nodes that are shared by a pair of overlapping modules. We find that nodes that lie in the overlap of the gold standard disease modules are mostly transcription factors (TF). Figure 7 shows the box plot of the distribution of the number of enriched modules the TFs are part of. Transcription factors regulate the expression of multiple genes and hence affect multiple pathways of varying functions. Since TFs control different functions, they are expected to be found in overlapping regions of the disease modules.

### 3.5. Overlapping Community Detection in the Absence of Known Disease Seed Nodes

As we established the importance of overlapping community detection for disease module identification it is also necessary to explore this approach in the absence of known disease seed nodes. We have selected HITS and spread hubs, which selects nodes based on their degree, to identify seed nodes. For each network, we kept the number of seed nodes fixed as for gold-standard disease module, and selected those many seed nodes using HITS and spread hub. The enriched and predicted modules obtained after seed node expansion can be found in the Table S4. This table also compares against the gold standard disease module results. It is observed that the best results in terms of the number of enriched modules predicted, is obtained for the PPI-1 network.

**Figure 8:**
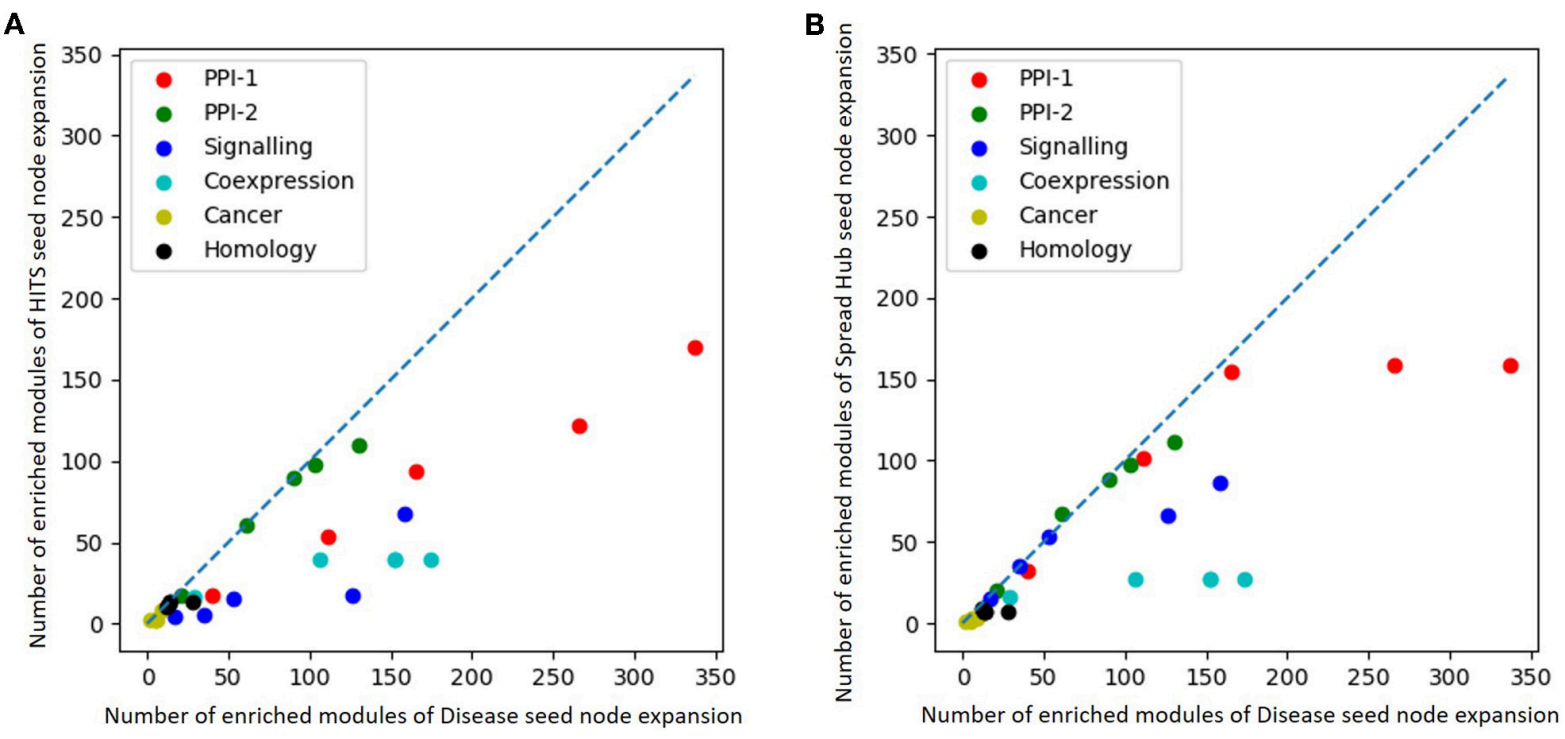
Scatter plot comparing number of enriched module predicted by **(A)** HITS **(B)** Spread hub seed node expansion against disease seed node expansion. The X and the Y axis represents the number of enriched modules disease seed node and unsupervised seed node expansion respectively. Different colored bubbles represents different network as mentioned in the legend. The partition line in the plot is x=y where performance of disease seed node expansion is similar to unsupervised seed node expansion.

The performance of unsupervised seed node expansion is visually compared with the gold standard module identification process with the help of scatter-plots as in Figure 8. The X-axis and the Y-axis of the plot represent the number of enriched modules as predicted by gold-standard module identification and unsupervised seed node expansion respectively. Different colors in the plots correspond to different networks as mentioned in the legend. For the same network, the plot shows multiple bubbles; those are with respect to the different number of seed nodes used for expansion. The line in the plot is for *x* = *y*, where the performance of disease seed node expansion is similar to unsupervised seed node expansion. As is quite intuitive, all the bubbles are below the partition line which means that the performance of disease seed node expansion is consistently better. It is observed that the performance of unsupervised seed node expansion on PPI-2 is comparable to its gold-standard disease module counterpart. Also, Figure 8B shows that the performance of spread hub as seed node selection is quite close to the disease seed node expansion as all the bubbles are much closer to the partition line.

#### 3.5.1. Sensitivity Analysis of Non-overlapping and Overlapping Clustering Approaches

The methods based on optimizing a “quality function,” such as conductance or modularity, non-overlapping communities, which means a node is part of a single module. The other class of methods we concern ourselves with are the non-overlapping clustering approaches using seed node based expansion methods. Below, we perform a detailed comparison of both classes of methods. We compare the number of enriched modules obtained from seed node based expansion methods with the quality function based approaches to understand the superiority of one over the other.

However, for a fair comparison between the approaches all of them should have a similar setup that is they should have either overlapping or non-overlapping communities. Therefore, we convert overlapping communities to non-overlapping communities using methods defined in section 2.4

The performance of the quality function-based community detection and seed node expansion methods are compared in the Table 5. The results suggest that identifying important nodes in the network and localizing communities around them is a better way of performing disease module identification, where compared to growing and merging communities from all possible nodes as done in quality function based approaches.

**Table 5:** Comparing quality function-based community detection with seed nodes expansion method after converting overlapping to non-overlapping communities for fair comparison.

**Network**	**Quality function based**		**Seed expansion based**		
	**Modularity**	**CEIL**	**Disease**	**HITS**	**Spread hub**
PPI 1	8 (262)	12 (1398)	26 (283)	24 (921)	28 (888)
PPI 2	9 (209)	11 (1696)	16 (191)	11 (659)	16 (207)
Signaling	10 (111)	6 (320)	15 (219)	10 (192)	12 (183)
Co-expression	10 (194)	5 (1336)	24 (234)	15 (743)	11 (240)
Cancer	4 (164)	5 (831)	11 (209)	6 (239)	7 (962)
Homology	6 (177)	7 (320)	9 (159)	3 (172)	9 (185)
**Total**	47	46	101	69	83

*The values in the table represent the number of enriched modules along with the predicted modules in the bracket*.

### 3.6. Overlapping Disease Modules Helps in Identifying Comorbid Diseases

**Figure 9:**
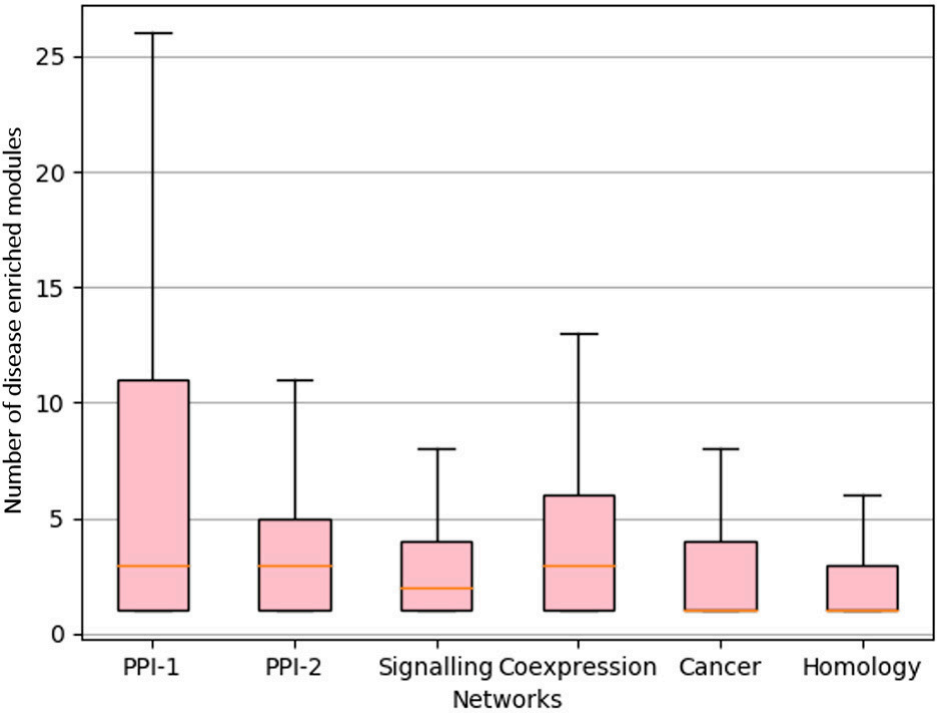
Distribution of number of GWAS datasets associated with the disease enriched module identified by various approaches. The X-axis represents different networks and Y-axis represents number of GWAS datasets associated with the disease module. The orange line represents the mean of the distribution.

We proceeded to derive useful insights from gold standard modules by studying comorbidity among diseases, i.e., those disease which have chances of co-occurring together. Comorbidity study is done by identifying diseases associated with the same disease enriched modules. Figure 9 shows a box-plot, representative of the distribution of the number of diseases that are associated with an enriched module; these modules are identified by various approaches which are already discussed in this and previous chapters. The distribution shows that there are multiple diseases associated with a disease enriched modules, especially in PPI-1. Modules enriched for multiple diseases are helpful in finding the association between the diseases. A module represents a group of genes responsible for diseases. Thus, if a person gets a particular disease due to improper functioning of few genes, then (s)he is likely to get another disease whose underlying responsible genes are the same. This study can help in answering questions such as, if a person has a particular disease, then how likely he can have another disease. As PPI-1 has the highest number of comorbid associations, we choose modules identified on this network for co-morbidity study.

**Figure 10:**
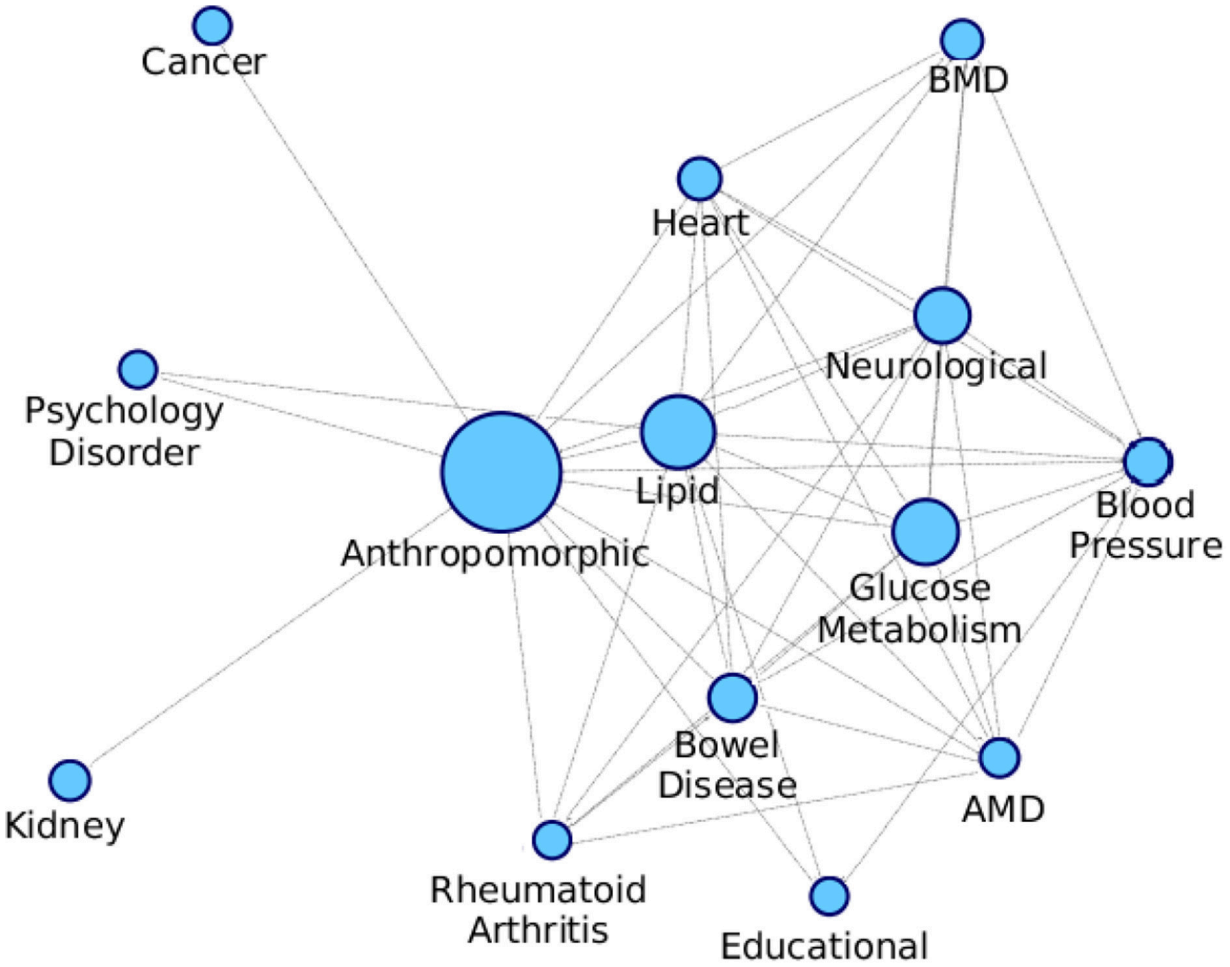
Comorbidity network showing associations between different diseases, created on the basis of number of GWAS datasets associated with enriched modules. The nodes are sized based on the number of GWAS datasets associated with the disease. AMD stands for Advanced Macular Disorder and BMD stands for Bone Mineral Density. These diseases are the same as those in Table S1. Hepatitis-C mentioned in the table is not in the comorbid network as there is minimal evidence of it being associated with any other disease.

We formed a comorbid network where the nodes are different diseases as shown in Table S1 and the edges are indicative of a module being enriched with the connected disease nodes. We consider the modules that are getting enriched with multiple diseases and connect all these diseases with an edge, and we also keep an edge count as to how many times those two diseases occurred together. Figure 10 shows the comorbid network created from the association between diseases of enriched modules on PPI-1, here we have kept top 50% associations based on the edge count.

The top associations represent the most frequently co-occurring diseases identified, based on the modules enriched with multiple diseases. Higher association between two disease means that there is more evidence for their correlation, as they have been grouped together more number of times. From Figure 10, anthropomorphic disease are seen to be connected with most of the other diseases suggesting that it is linked with many of the diseases. Further, the links between Glucose Metabolism and Lipid/Heart are also not very surprising, given the remarkable co-ocurrences of diabetes, coronary heart diseases, and hypercholesterolemia etc.

## 4. Discussion

The identification of communities in networks is a well-studied problem in computer science. In this DREAM challenge, the goal was to identify such modules, or communities, in various biological networks, and study their association with diseases. In the present study, we examined various approaches for community detection, and their applicability to biological networks, to identify disease-relevant modules. Notably, we illustrate the importance of identifying smaller “core” communities compared to standard non-overlapping clustering algorithms. Further, we analyse the need and importance of overlapping communities and the utility of seed nodes or partial knowledge in greatly improving the prediction of biological relevant disease modules from diverse networks.

We have three key results. First, we show that well-known non-overlapping clustering approaches fail to identify sufficient number of relevant disease modules. Our core-module based identification methods, which identify smaller and structurally better communities, could identify larger number of disease-enriched modules than those identified by well-known non-overlapping community detection approaches. The state-of-the-art non-overlapping clustering approaches detect large communities and the core module identification approaches detect small communities as can be seen in the Figure S2. In almost all the cases, we saw an improvement in the performance on downsizing the size of the communities. It is important to note that this was also affected by the DREAM challenge evaluation, which mandated the identification of smaller communities ranging from 3–100 nodes. Nevertheless, such smaller communities are more common in biological networks (Wilber et al., 2009), and can indeed capture more disease-relevant communities as observed in the results. Another important observation was that the different networks provided in the challenge present diverse views of the interactions happening in the cell. Therefore, each network has different network properties and consequently need different approaches to identify disease modules in them. For example, PPI-1 had smaller-sized disease modules than PPI-2 as can be observed in Figure 9, and hence *Multiple Core Identification* was able to perform better than *MCL* as the former method downsizes the size of the community. *Min Outgoing edges* further reduced the size of the module thus improving on the number of modules identified. For signaling and co-expression network we know that genes interacting with more number of other genes are biologically more active (Vidal et al., 2011) and this could be seen in the results – method *min outgoing edges* which gives more importance to nodes with higher degree showed a remarkable improvement over *modularity maximization* method. It was hard to achieve good performance for cancer network as despite using known disease nodes in Table 3, module identification gave very poor number of disease enriched modules. For the homology network, ensemble with *min outgoing edges* was useful. Overall, the number of disease-enriched modules identified by core module-based methods was higher than those identified by the baseline approaches. The reason for performance improvement on applying the proposed heuristics is due to the identification of core modules, which are smaller and structurally more informative.

Second, we investigated the clusterability properties of disease modules and illustrated that there do exist well-defined communities, but a overlapping clustering approach was important to capture them, particularly in face of the fact that most proteins have multiple functionalities or cause different diseases. Owing to the lack of ground truth disease module, the enriched modules identified after exploiting domain knowledge—the “gold standard modules”—have better cluster quality than their non-enriched counterparts. This indicates that disease modules, when carefully identified with the help of some known disease nodes, possess good clusterability.

Third, we showed that information on “seed nodes” underlying these modules can substantially improve the identification of disease-relevant modules. As the fraction of disease seed node increases number of identified disease enriched modules increases (Table 4). Interestingly when disease nodes are not known identifying seed nodes using spread-hubs and doing seed expansion on it perform equally well especially for lower fraction of seed nodes as can be seen in Table S4. Thus further supporting the fact, overlapping community detection is a better way to identify disease modules. Also, the overlaps between gold standard module identification are also useful for identifying co-occurring diseases, such that occurrence of one disease results is a signal that the other one can also occur. We also show that localizing community discovery around a network-centric, biologically relevant node (seed node) offers a clear advantage for disease module identification in comparison with a completely unsupervised approach. Domain guidance is essential and should be leveraged upon whenever possible. We observe this when one compares the performance of quality function based methods with the seed expansion strategy than extant approaches as in Table 5.

Our study underlines the need to develop biologically motivated clustering algorithms that are able to better capture “disease community structure” and notably, emphasizes the importance of overlapping clustering approaches to reliably identify disease-relevant modules and comorbidity networks from diverse biological datasets. Notably, our results underline the importance of overlapping community detection and makes the case for further investigation into such methods, rather than non-overlapping community identification, in the case of biological networks.

## Data Availability

The datasets and codes for this study are available on GitHub: https://github.com/RamanLab/DiseaseModuleIdentification.

## Author Contributions

BT, KR, and BR designed the experiments. BT performed the experiments. BT, SP, KR, BR, and HS analyzed the results and wrote the manuscript.

### Conflict of Interest Statement

The authors declare that the research was conducted in the absence of any commercial or financial relationships that could be construed as a potential conflict of interest.

## Footnotes

^1^https://www.synapse.org/#!Synapse:syn6156761/wiki/400645
