# Supplementary material for: Adapting Community Detection Algorithms for Disease Module Identification in Heterogeneous Biological Networks
Source: Front Genet. 2019 Mar 13;10:164. doi: 10.3389/fgene.2019.00164 (PMC6424898; doi:10.3389/fgene.2019.00164)
Supplement: Supplementary file 1 [file Data_Sheet_1.pdf]

Supplementary Material: Adapting Community Detection Algorithms for Disease Module Identification in Heterogeneous Biological Networks

1 SUPPLEMENTARY FIGURES

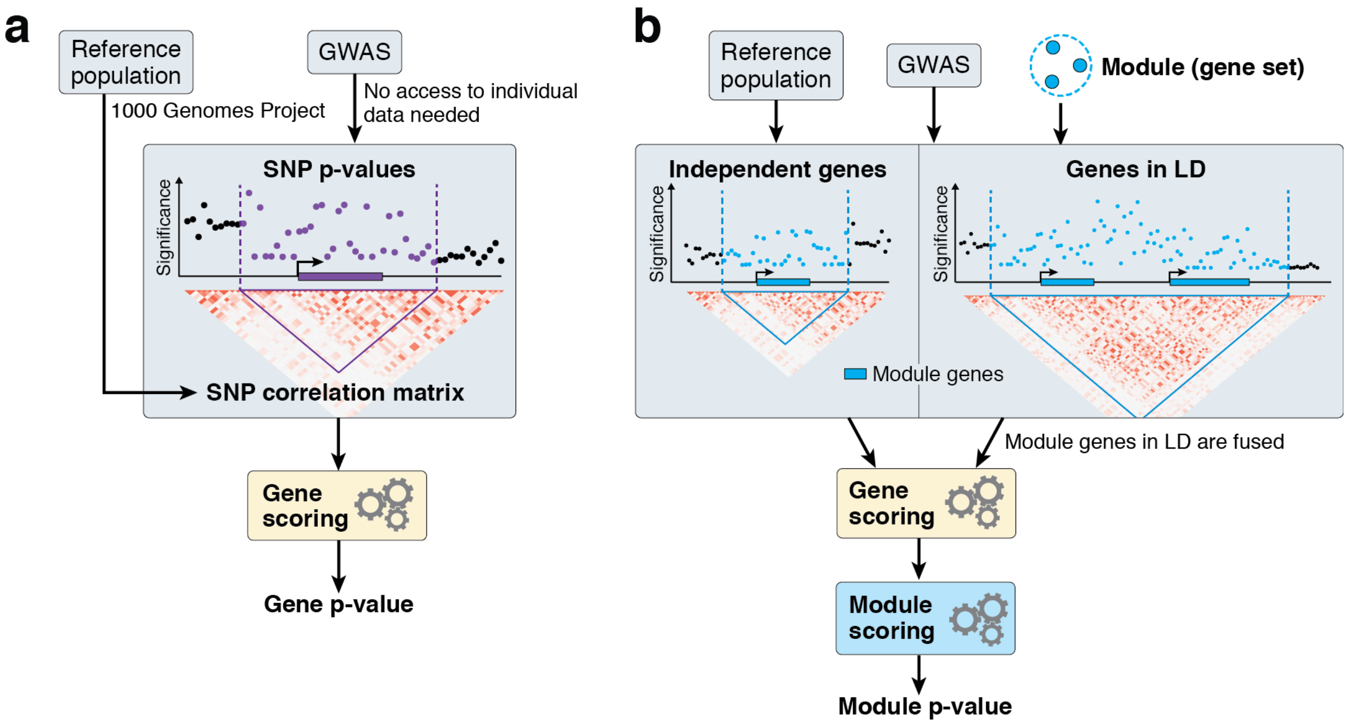

Figure S1: PASCAL scoring tool for computing module enrichment score. This image is licensed under the CC BY 4.0 license and attributed to the synapse website (challenge web-page): `https://www.synapse.org/#!Synapse:syn6156761/wiki/401425`.

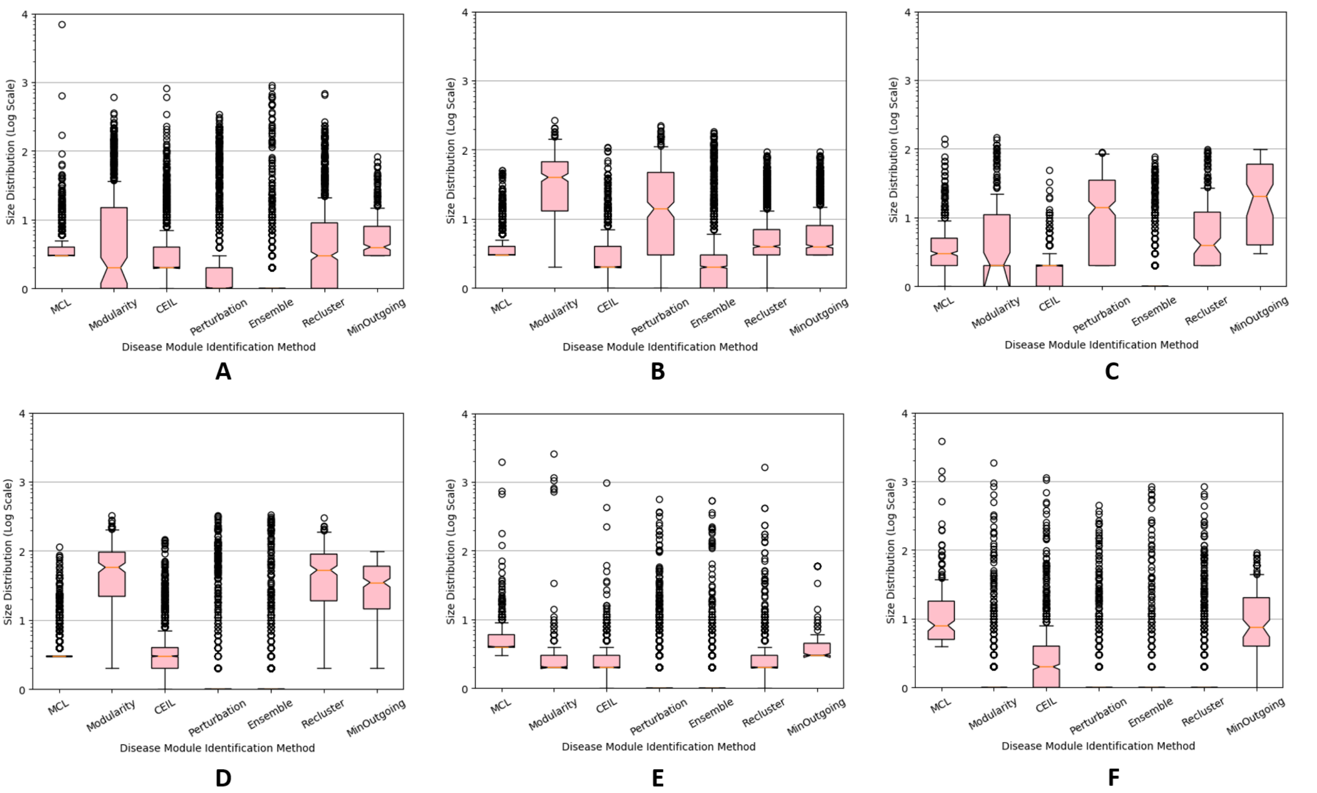

Figure S2: Size distribution of modules generated by non-overlapping community detection approaches on (A) PPI-1, (B) PPI-2, (C) Signalling, (D) Coexpression, (E) Cancer and (F) Homology network.

2 SUPPLEMENTARY TABLES

Table S1. 180 GWAS datasets into 15 broader categories of diseases

GWAS Groups		
Anthropomorphic	Lipid	Heart
Neurological	Bone Mineral Density	Rheumatoid Arthritis
Bowel Disease	Psychology Disorder	Cancer
Glucose Metabolism	Educational related disorders	Hepatitis-C
Advanced Macular Disorder	Blood Pressure	Kidney

Table S2. Hyper-parameter of module identification on simple networks, using off-the-shelf approaches mentioned as baselines and core module identification proposed by us. Hyper-parameter for MCL is inflation (I), modularity maximisation is resistance (R), and all the core module identification methods are applied on baseline method — so the method and their appropriate parameter are shown.

**Method**	**Baselines**			**Core Module based Methods**			
	**MCL**	**Modularity Maximization**	**CEIL**	**Perturbation**	**Ensemble**	**Recluster**	**Min Outgoing**
**Parameter**	Inflation (I)	Resistance (R)	None	Method (Parameter)	Method (Parameter)	Method (Parameter)	None
**PPI 1**	6	0.1	-	Modularity (R=0.1)	All	MCL (I=3)	-
**PPI 2**	7	0.9	-	Modularity (R=0.1)	All	MCL (I=7)	-
**Signaling**	2	0.2	-	Modularity (R=0.1)	All	Modularity (R=0.4)	-
**Coexpression**	3	0.1	-	Modularity (R=0.1)	All	Modularity (R=0.1)	-
**Cancer**	2	0.4	-	Modularity (R=0.1)	All	Modularity (R=0.8)	-
**Homology**	2	0.1	-	Modularity (R=0.1)	All	Modularity (R=0.2)	-

Table S3. Baseline methods are tuned over their hyper-parameters which are resistance for Modularity and Inflation for MCl, there is no parameter for CEIL. The values shown represent the number of enriched modules and the total number of modules predicted in brackets. The best results are marked in bold and their corresponding hyper-parameter are present in Table S2.

Parameter	PPI-1	PPI-2	Signalling	Co expression	Cancer	Homology
Modularity (Resistance)						
0.1	**8 (262)**	**9 (209)**	10 (178)	**10 (194)**	2 (596)	6 (177)
0.2	7 (69)	5 (61)	**10 (111)**	9 (30)	3 (454)	**10 (134)**
0.3	5 (8)	3 (31)	8 (87)	4 (5)	4 (308)	5 (79)
0.4	3 (3)	5 (17)	4 (59)	1 (1)	4 (164)	4 (51)
0.5	3 (3)	3 (13)	4 (49)	0 (0)	5 (82)	7 (29)
0.6	2 (6)	4 (13)	4 (49)	0 (0)	7 (52)	5 (20)
0.7	3 (4)	7 (15)	2 (43)	0 (0)	**7 (30)**	5 (19)
0.8	4 (4)	3 (13)	2 (38)	0 (0)	3 (26)	7 (23)
0.9	2 (2)	3 (12)	1 (36)	0 (0)	7 (19)	7 (22)
1	3 (4)	3 (13)	1 (30)	0 (0)	4 (22)	5 (21)
MCL (Inflation)						
2	12 (152)	10 (1259)	**9 (268)**	8 (525)	**4 (598)**	**8 (180)**
3	**17 (547)**	15 (1367)	8 (333)	**9 (463)**	3 (640)	6 (253)
4	14 (738)	16 (1273)	7 (339)	7 (353)	3 (658)	6 (321)
5	15 (822)	15 (1195)	6 (342)	7 (293)	3 (664)	4 (364)
6	16 (872)	18 (1125)	7 (337)	6 (251)	1 (666)	5 (409)
7	14 (857)	17 (1079)	7 (337)	5 (223)	1 (678)	4 (445)
8	14 (859)	**17 (1039)**	6 (335)	4 (208)	1 (666)	4 (462)
9	12 (870)	13 (1010)	6 (332)	7 (197)	2 (664)	2 (479)
CEIL						
-	12 (1398)	11 (1696)	6 (320)	5 (1336)	5(831)	7(320)

Table S4. Comparing unsupervised seed node expansion with known seed node expansion across networks, with the number of seed nodes decided on the basis of disease seed node p-value cut-off. The values shown represent the average number of enriched modules and the total number of modules predicted, averaged across five runs.

p-value		# Seed	Gold	HITS	Spread
Fraction	Cut-off	Nodes	Standard		Hub
**PPI-1 network**					
100%	10^{-4}	5436	337 (5433)	170 (4055)	159 (2648)
100%	10^{-6}	3103	266 (3101)	122 (3101)	159 (2648)
80%	10^{-6}	2482	165.8 (2147.6)	94 (2480)	155 (2477)
50%	10^{-6}	1551	111 (1378.4)	54 (1549)	101 (1550)
10%	10^{-6}	310	40 (292.8)	17 (309)	32 (309)
**PPI-2 network**					
100%	10^{-4}	3876	130 (3844)	110 (3861)	112 (3829)
100%	10^{-6}	2267	103 (2250)	98 (2264)	97 (2266)
80%	10^{-6}	1813	90 (1357.2)	90 (1812)	88 (1813)
50%	10^{-6}	1133	60.6 (897.4)	61 (1133)	67 (1133)
10%	10^{-6}	226	20.6 (202.6)	17 (226)	20 (226)
**Signalling Network**					
100%	10^{-4}	1893	158 (1840)	68 (1893)	86 (1834)
100%	10^{-6}	1174	126 (1139)	17 (916)	66 (1174)
80%	10^{-6}	939	53.4 (597.6)	15 (939)	53 (939)
50%	10^{-6}	587	35.2 (407.6)	5 (587)	35 (587)
10%	10^{-6}	117	16.8 (98.2)	4 (117)	15 (117)
**Co-expression Network**					
100%	10^{-4}	4099	174 (4094)	40 (1064)	27 (1008)
100%	10^{-6}	2406	152 (2404)	40 (1064)	27 (1008)
80%	10^{-6}	1924	152.2 (1859.4)	40 (1064)	27 (1008)
50%	10^{-6}	1203	105.8 (1167.4)	40 (1064)	27 (1008)
10%	10^{-6}	240	29 (236)	16 (240)	16 (240)
**Cancer Network**					
100%	10^{-4}	4507	6 (4429)	2 (4487)	3 (4417)
100%	10^{-6}	2555	2 (2522)	2 (2538)	1 (2555)
80%	10^{-6}	2044	5 (1484.2)	2 (2030)	1 (2044)
50%	10^{-6}	1277	5.2 (979.6)	2 (1273)	2 (1277)
10%	10^{-6}	255	8.4 (216.8)	8 (255)	3 (255)
**Homology Network**					
100%	10^{-4}	3227	28 (3154)	13 (708)	7 (618)
100%	10^{-6}	1861	14 (1826)	13 (708)	7 (618)
80%	10^{-6}	1488	14.2 (1221.8)	13 (708)	7 (618)
50%	10^{-6}	930	13 (793.8)	11 (708)	7 (618)
10%	10^{-6}	186	12 (174.2)	10 (185)	9 (186)
